# Application of Biotechnology for the Production of Biomass-Based Fuels

**DOI:** 10.1155/2017/3896505

**Published:** 2017-05-23

**Authors:** Liandong Zhu, Ningbo Gao, Rong-Gang Cong

**Affiliations:** ^1^Department of Energy Technology, University of Vaasa, 65101 Vaasa, Finland; ^2^School of Energy and Power Engineering, Xi'an Jiaotong University, Xi'an 710049, China; ^3^Department of Environmental Science, Aarhus University, 4000 Roskilde, Denmark

In response to the energy crisis, global warming, and climate changes, biomass has received a great deal of interest as a promising feedstock for the production of biofuels. Biofuels derived from biomass are renewable and sustainable energies with the potential to replace fossil fuels. In addition, the development of biofuels might reduce a country's reliance on crude oil imports, mitigate greenhouse gas emissions, and increase regional incomes. To determine the stand of the latest available biotechnologies and keep the global academic communities up to date to the current advances in the conversion of biomass to biofuels, this special issue is publishing 10 quality papers with the focus on the application of biotechnology for the production of biomass-based fuels.

The paper titled “Isoprene Production on Enzymatic Hydrolysate of Peanut Hull Using Different Pretreatment Methods” by S. Wang et al. described the work on the use of peanut hull for isoprene production. The authors applied two pretreatment methods, hydrogen peroxide-acetic acid (HPAC) and popping, prior to enzymatic hydrolysis. Results demonstrated that the isoprene production on enzymatic hydrolysate with HPAC pretreatment was about 1.9-fold higher than that of popping pretreatment. The amount and category of inhibitors such as formic acid, acetic acid, and HMF varied among different enzymatic hydrolysates. In addition, results showed that the enzymatic hydrolysate of HPAC was detoxified by activated carbon.

The paper titled “Studies on the Ecological Adaptability of Growing Rice with Floating Bed on the Dilute Biogas Slurry” by Q. Kang et al. assessed the adaptability and possibility of growing rice on floating beds with diluted biogas slurry. The authors discovered that the growth stage, rice plant height, and rice yield and quality were significantly affected by multiple dilutions. It is found that the rice plants cultivated with 45 multiple dilutions had better ecological adaptability than others. Their study showed that it is possible and safe to cultivate rice plants with diluted biogas slurry. The yield, milled rice rate, and crude protein of the rice cultivated with slurry are not as much as those of rice cultivated with regular way in soil.

The paper titled “Strategies for Lipid Production Improvement in Microalgae as a Biodiesel Feedstock” by L. D. Zhu et al. presents a review on the application of the strategies to activate lipid accumulation, which opens the door for lipid overproduction in microalgae. The review highlights the main approaches for microalgal lipid accumulation induction to expedite the application of microalgal biodiesel as an alternative to fossil diesel for sustainable environment. To promote microalgal biodiesel production during the scale-up process, the achievement of lipid overproduction is essential, and certain appropriate strategies can help realize the goal. But, in practice, the lipid-inducing strategies can also be combined in an effort to achieve lipid production optimization.

The paper titled “Simultaneous Saccharification and Fermentation of Sugar Beet Pulp for Efficient Bioethanol Production” by J. Berłowska et al. explored an approach to investigate the effects of pretreatment, the dosage of cellulase and hemicellulase enzyme, and aeration on the release of fermentable sugars and ethanol yield during the simultaneous saccharification and fermentation (SSF) of sugar beet pulp-based worts. The results showed that a 6 h interval for enzymatic activation between the application of enzyme preparations and inoculation with ethanol red could further improve the fermentation performances, with the highest ethanol concentration reaching 26.9 g/L and 86.5% fermentation, compared to the theoretical yield.

The paper titled “Application of the Initial Rate Method in Anaerobic Digestion of Kitchen Waste” by L. Feng et al. developed a method of methane production through the determination of the hydrolysis constants and reaction orders at both low total solid (TS) concentrations and high TS concentrations. The results showed that the first-order hydrolysis model better reflected the kinetic process of gas production. During the experiment, all the influential factors of anaerobic fermentation retained their optimal values. For a long reaction time, the authors believed that the hydrolysis involved in anaerobic fermentation of kitchen waste could be regarded as a first-order reaction in terms of reaction kinetics.

The paper titled “Outdoor Growth Characterization of an Unknown Microalga Screened from Contaminated Chlorella Culture” by S. Huo et al. used 18 s rDNA molecular technology to isolate and identify one wild strain* Scenedesmus* sp. F. S. from the culture of* Chlorella zofingiensis*. The authors discovered that* Scenedesmus* sp. F. S. showed good alkali resistance and robust adaption to the stress of the outdoor environment. Furthermore, under normal conditions, the oil content of* Scenedesmus* sp. F. S. could reach more than 22.0%, and C16–C18 content could reach up to 79.7%, showing that it had great potential as a large-scale cultivation strain for biodiesel production.

The paper authored by C. Quan and N. Gao provides a review on the copyrolysis of coal and biomass and then compares their results with those obtained using coal and biomass pyrolysis in detail. They also discuss the effects of reaction parameters such as feedstock types, blending ratio, heating rate, temperature, and reactor types on the occurrence of synergy and point out the main properties of the copyrolytic products.

The paper written by K. T. Dasa et al. examined the inhibitory effects of LCFAs (palmitic, stearic, and oleic acid) on biogas production as well as the protective effect of a membrane bioreactor (MBR) against LCFAs. Their findings showed that palmitic and oleic acid with concentrations of 3.0 and 4.5 g/L resulted in >50% inhibition on the biogas production, while stearic acid had an even stronger inhibitory effect. The encased cells in the MBR system were found to be able to perform better in the presence of LCFAs.

The paper authored by I. Masin and M. Petru dealt with the complex approach to design specification that could bring new innovative concepts to the design of mechanical machines for oil extraction. Their presented case study as the main part of the paper focused on the new concept of the screw of machine mechanically extracting oil from* Jatropha curcas* L. seeds.

The paper written by Wang et al. conducted the FTIR analysis which showed that the chemical structure of lignin was broken down in the LHW process. In addition, they also explored the impact of untreated and treated lignin on the enzymatic hydrolysis of cellulose. They also found that the LHW-treated lignin had little impact on the cellulase adsorption and enzyme activities and somehow could improve the enzymatic hydrolysis of cellulose.

